# Breaking Barriers to Healthcare Access: A Multilevel Analysis of Individual- and Community-Level Factors Affecting Women’s Access to Healthcare Services in Benin

**DOI:** 10.3390/ijerph18020750

**Published:** 2021-01-17

**Authors:** Betregiorgis Zegeye, Ziad El-Khatib, Edward Kwabena Ameyaw, Abdul-Aziz Seidu, Bright Opoku Ahinkorah, Mpho Keetile, Sanni Yaya

**Affiliations:** 1 HaSET Maternal and Child Health Research Program, Shewarobit Field Office, Shewarobit P.O. Box 127, Ethiopia; betregiorgiszegeye27@gmail.com; 2 Department of Global Public Health, Karolinska Institutet, SE-171 77 Stockholm, Sweden; ziad.el-khatib@ki.se; 3 Medical University of Vienna, Vienna 1090, Austria; 4 World Health Programme, Université du Québec en Abitibi-Témiscamingue (UQAT), Rouyn-Noranda, QC J9L 2K1, Canada; 5 School of Public Health, Faculty of Health, University of Technology Sydney, Sydney, NSW 2007, Australia; edmeyaw19@gmail.com (E.K.A.); brightahinkorah@gmail.com (B.O.A.); 6 Department of Population and Health, University of Cape Coast, Cape Coast, PMB 0494, Ghana; abdul-aziz.seidu@stu.ucc.edu.gh; 7 College of Public Health, Medical and Veterinary Sciences, James Cook University, Townsville, QLD 4811, Australia; 8 Department of Population Studies, Faculty of Social Sciences, University of Botswana, Private Bag UB 0022, Gaborone, Botswana; mphokeet@yahoo.com; 9 School of International Development and Global Studies, University of Ottawa, Ottawa, ON K1N 6N5, Canada; 10 The George Institute for Global Health, Imperial College London, London W12 0BZ, UK

**Keywords:** access, healthcare services, barriers, risk factors, global health, reproductive health

## Abstract

*Background:* In low-income countries such as Benin, most people have poor access to healthcare services. There is scarcity of evidence about barriers to accessing healthcare services in Benin. Therefore, we examined the magnitude of the problem of access to healthcare services and its associated factors. *Methods:* We utilized data from the 2017–2018 Benin Demographic and Health Survey (n = 15,928). We examined the associations between the demographic and socioeconomic characteristics of women using multilevel logistic regression. The outcome variable for the study was problem of access to healthcare service. Adjusted odds ratios (AORs) with 95% confidence intervals (95% CI) were estimated. *Results:* Overall, 60.4% of surveyed women had problems in accessing healthcare services. Partner’s education (AOR = 0.70; 95% CI; 0.55–0.89), economic status (AOR = 0.59; 95% CI; 0.47–0.73), marital status (AOR = 0.44; 95% CI; 0.39–0.51), and parity (AOR = 1.85; 95% CI; 1.45–2.35) were significant individual-level factors associated with problem of access to healthcare. Region (AOR = 5.24; 95% CI; 3.18–8.64) and community literacy level (AOR = 0.69; 95% CI; 0.51–0.94) were the main community-level risk factors. *Conclusions:* Enhancing husband education through adult education programs, economic empowerment of women, enhancing national education coverage, and providing priority for unmarried and multipara women need to be considered. Additionally, there is the need to ensure equity-based access to healthcare services across regions.

## 1. Introduction

Health is vital for having a socially and economically productive life [1]. The health and wellbeing of adults are critical for maintaining the welfare of the household, including children, as they directly affect the capacity to work [1]. Healthcare access affects an individual’s entire health condition, such as physical, mental, and social, as well as overall quality of life [2]. Obtaining access to holistic and high-quality care is essential for achieving and maintaining good health, averting and managing diseases, subsiding the likelihood of infirmity and untimely death, and realizing equity in health [2]. According to the World Health Organization (WHO), healthcare service is every citizen’s basic human right, and it is the duty of the country to ensure that healthcare services are acceptable, accessible, and timely [3].

Healthcare accessibility has multiple dimensions and is affected by accessibility and availability of service and quality of service given at health facility, as well as geographical and financial accessibility [3]. The utilization of health services is mainly related to accessibility of healthcare [4,5]. Worldwide, nearly 400 million people lack healthcare access, and eight million people die due to treatable health problems, which again leads to approximately six trillion USD economic loss in low- and middle-income countries [6]. Globally, 150 million people suffer financial crisis related to payment for healthcare services [7]. In 2015, the global leaders approved the Sustainable Development Goals (SDGs) and committed to accomplish universality in health coverage comprising access to affordable and quality essential medicines and financial protections [8]. Related to this, most of the countries in sub-Saharan Africa have universal health coverage as one of the national health strategies. However, the trend of changing this commitment into visible output through mobilization of national budgets in the areas of health, financial protection, and equitable and quality health services are still low [9].

In the Benin constitution, it is recorded that the concept of health is a human right [10]. Until the impartial financial protection and healthcare accesses are addressed, it is difficult to admit that universal health coverage (UHC) is ensured [10]. Impartial financial protection is that each person, regardless of socioeconomic status, does not encounter severing financial adversity related to essential health services [10]. 

The Benin health system emphasizes the public subdivision, with a history of strong governing or regulatory processes, and central executive or decision-making authority [10,11]. The Benin national health development plan is segmented into triennial development plans so as to obviate barriers and advance governance and health resources’ administration [10]. In Benin, out of the 34 health zones, 30 are fully functional, and the health coverage in the country is 77%. However, unfairness exists in the delivery of health facilities, with rural areas getting less healthcare services compared to urban settings [10,12]. A large portion of Beninese have poor access to health services [10,13]. About 37.7% of their health expenditures are out-of-pocket (OOP), and the majority of OOP health expenditure is made in the private sector [13].

Barriers to accessing healthcare services generate a situation where health needs of the people are not fully met or there is a failure to have healthcare, leading to financial burden and unnecessary admission or hospitalization [2]. Providing great attention both on coverage and barriers to accessing healthcare services is vital for public health researchers to provide input for policy makers to evaluate the present policy, programs, and interventions of health service access and to redesign or improve them [14].

Evidence show that socioeconomic factors, such as women and their husbands’ educational level, household economic status, place of residence, language barriers, occupational status, and autonomy of women, affect women’s access to health services [15,16]. However, there is a dearth of evidence in Benin relating to access to health services. Therefore, we examined the factors associated with access to healthcare services in Benin.

## 2. Materials and Methods

### 2.1. Sources of Data and Sampling Procedure

We used data from the 2017–2018 Benin Demographic and Health Survey (BDHS) [17]. It aims to provide up-to-date information for monitoring the health situation in Benin. It is conducted in collaboration with the financial and technical help of Inner-City Fund (ICF) and the Demographic and Health Survey (DHS) program that is funded by the United States Agency for International Development (USAID). It is a nationally representative survey that aims to collect data on many topics, including barriers to healthcare access. It is a standard survey normally carried out in two stages [17]. The 2017–2018 BDHS included 14,156 households; interviewed 15,928 women in the reproductive age groups (15–49 years) and 7595 men aged 15–59; 13,589 and 13,643 unweighted and weighted children < 60 months, respectively [17]. 

The BDHS was designed using two-stage stratified cluster sampling. In the first stage, using the probability proportional to size (PPS) technique, enumeration area was selected, which is a large geographic area that includes many households. Housing listing was done in each enumeration area before households were selected in order to prepare sampling frame. Then, fixed numbers of households were selected from each enumeration area in the second stage. Regarding overall BDHS methods, it has been discussed and is available in the 2017–2018 BDHS final French report, and readers can consult it for further understanding [17]. We used the file that contains survey women’s data, individual recode (IR file) (https://dhsprogram.com/data/dataset/Benin_Standard-DHS_2017.cfm), for analysis. The dataset is freely available to the public through https://dhsprogram.com/data/dataset/Benin_Standard-DHS_2017.cfm?flag=0 [18]. 

### 2.2. Variables

#### Dependent Variable

The problem of access to healthcare services was the dependent variable of this study. In the 2017–2018 Benin DHS, all women were asked the following four questions to measure access to healthcare services: “Do you think the following four reasons are big problems when you become sick and seek medical treatment or advice?” (1) Getting approval to go to the health facility? (2) Having money required for treatment or advice? (3) Distance from their home to health facility? (4) Inconvenient or not have desire to go alone?” The outcome variable was categorized by recoding “yes” responses to the items if they had a big problem in “getting permission to go to the doctor”, “getting money for advice or treatment”, “distance to health facility”, and “not wanting to go alone”. If they had no big problem, it was coded as “no”. Women who encountered at least one of those problems in these four areas were categorized as having a problem in accessing healthcare, while those who did not encounter any problem in the four domains were categorized as having no problem. 

### 2.3. Explanatory Variables

By referring to previous literature, several individual- and community-level factors were incorporated for their significant link to healthcare service access and utilization [19,20,21,22,23,24]. Included individual-level factors were age in years (15–19, 20–24, 25–29, 30–34, 35–39, 40–44, 45–49), women’s educational status (no formal education, primary, secondary, higher), husband’s educational status (no formal education, primary school, secondary school, higher), women’s employment/occupation (not working, professional/technical/managerial, sales, agricultural—self-employed, agricultural—employee, services, skilled manual, other unclassified), husband’s occupation (not working, professional or technical or managerial, sales, agricultural—self-employed, agricultural—employee, services, skilled manual, other unclassified), religion (Vodoun, Catholic, Islam, Protestant Methodist, other Protestants, Celestes, other Christians, other religions, no religion), wealth quintiles (poorest, poorer, middle, richer, richest), ethnicity (Adja and related, Bariba and related, Dendi and related, Fon and related, Yoa, lokpa and related, Betamaribe and related, Peulh and related, Yoruba and related, other Beninois, other nationalities), marital status (not currently married, married) and parity (0, 1–2, 3–4, ≥5). Community-level factors included were place of residence (urban or rural), region (Alibori, Atlantic, Atacora, Borgou, Couffo, Collines, Littoral, Donga, Mono, Oueme, Plateau, Zou), community literacy level (low, medium, high), and community socioeconomic level (low, moderate, high).

### 2.4. Statistical Analysis

We conducted the data analyses in this order: first, we conducted descriptive analysis (frequency distribution of participants and prevalence of healthcare access problem); second, we did bivariate analysis (chi-square test) to examine whether or not each explanatory variable had significant statistical association with the response variable using a *p*-value of <0.05 as a cut-off point. Next, we conducted a multi-collinearity test, using variance inflation factor (VIF) for all explanatory variables that had statistically significant association with the outcome variable, and found that there was no indication of high collinearity among the independent variables (mean VIF = 3.55, min = 1.14, max = 5.58). Evidence shows that VIF < 10 are tolerable [25,26]. Fourth, we conducted a multilevel logistic regression analysis by constructing four models: (a) an empty model (called model 0), as the first model, which emphasizes the variance in the response variable (healthcare access problem), accredited to the clustering at the primary sampling units (PSUs); (b) then we constructed model 1, to measure the individual-level factors that had associations with healthcare access problem; (c) then we constructed model 2, which included the community-level factors to ascertain their association with healthcare access problem; (d) finally, we constructed the full model (called model 3) that included the individual- and community-level factors. The multilevel logistic regression model consisted of random and fixed effects [27,28,29].

The fixed effects, also called measures of association, demonstrate results of the association between the independent variables and the dependent variable, and were stated as adjusted odds ratios (AOR) with their 95% confidence intervals (CIs), whereas the random effects, also called measures of variations, were measured with intra-cluster correlation (ICC) [29,30]. Likelihood ratio (LR) test was applied to confirm the adequacy of model. Model fitness or fitness of different models were checked using Akaike’s information criterion (AIC) and Bayesian information criterion (BIC) techniques. To take care for the complex structure of the data, we used the “svyset” command in the model so that all the three pieces of design elements (weight, cluster, and strata) would be taken into consideration. This procedure safeguards against the problem of inflated type one error and large CI at the same time. We used Stata version 14.0 (StataCorp, College Station, TX, USA).

### 2.5. Ethical Consideration 

For the analysis of this study, we used DHS data, which is publicly available. The DHS program is dependable, with standards for guaranteeing the safeguard of participants’ or respondents’ privacy. ICF International confirms that the survey conforms to the U.S. Department of Health and Human Services rules for respecting of human subjects’ rights. No additional consent was necessary for this study because the data is secondary and could be accessed in the public domain (https://dhsprogram.com/data/available-datasets.cfm) [31]. For more details of ethical issues related to data, please refer to http://goo.gl/ny8T6X [32].

## 3. Results

In total, we included 15,928 participants, where 21% were within the age groups of 15–19 years. Over half of each of the women (55%) and their husbands (56.6%) had no formal education. About 29.4% of the participants were Muslims, followed by 24.7% Catholics, whilst 36% of the participants were from Fon and related ethnic groups. Close to 55.7% of the participants were rural residents, and 26.3% of participants had five and above births (Supplementary Material 1).

**Figure 1:**
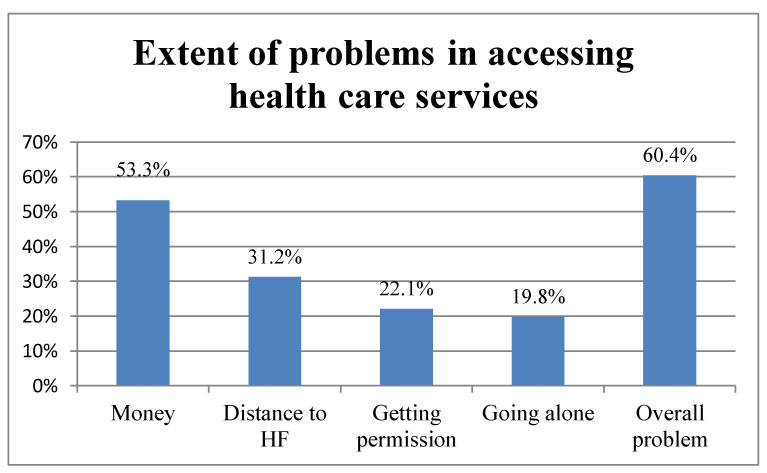
Extent of problems in accessing healthcare services among women in Benin: Evidence from 2017/2018 Benin Demographic and Health Survey (DHS).

Overall, 60.4% of women in the reproductive age group had problems in accessing healthcare (at least for one of the reasons of either getting permission, going alone, distance to health facility, or getting money for medical prescription). Money barrier was the first problem, and distance to health facility ranked second (Figure 1).

**Figure 2:**
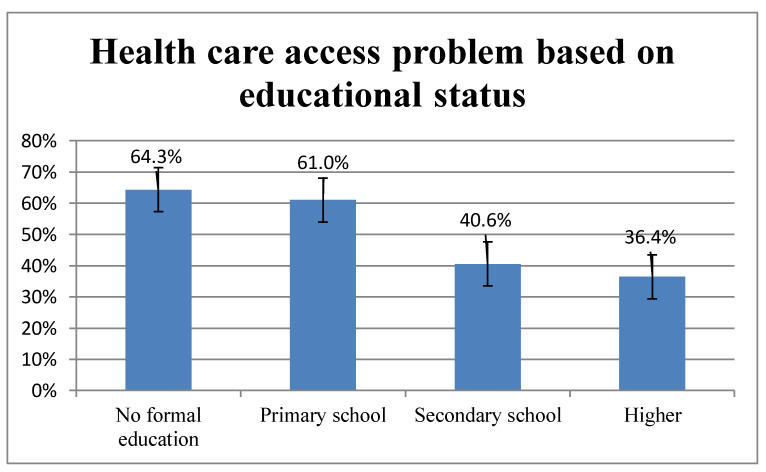
Magnitude of healthcare access problem among women of reproductive age (15–49) in Benin, based on educational status: evidence from 2018/2019 Benin DHS.

Problems of accessing healthcare profoundly varied across different subgroups of population (Supplementary Material 2): 64.3% of women without formal education had problems in accessing healthcare, and it decreased to 36.4% among women who had attended higher education (Figure 2). Similarly, partners of 37% of the women had higher education, whilst partners of 63% had no formal education. 

**Figure 3:**
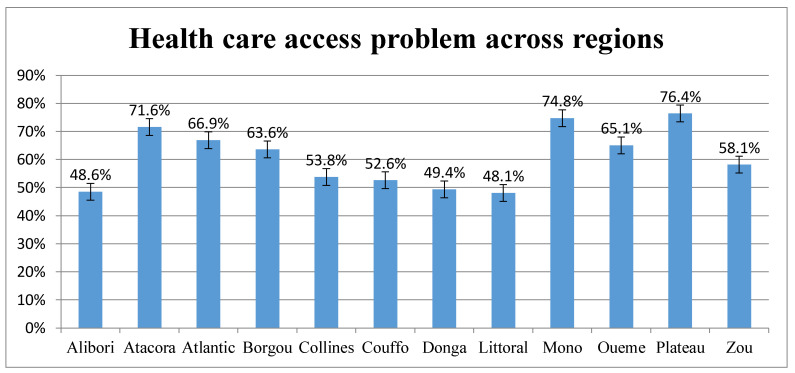
Magnitude of healthcare access problems among women of reproductive age (15–49) in Benin across regions: Evidence from 2018/2019 Benin DHS.

While 76% of women from Betamaribe and related ethnic groups had problems in accessing healthcare services, the magnitude declined to 43.3% among other nationalities. Problems of healthcare access also extensively varied across regions. For instance, about 48% of women residing in the Littoral region had problems in healthcare access. However, the problem increased to 76.4% among those in the Plateau region (Figure 3). 

### Measures of Associations

The individual-level predictors of barriers to healthcare access were husbands’ education (higher AOR = 0.70; 95% CI; 0.55–0.89), women’s occupation (agricultural—self-employed AOR = 1.28; 95% CI; 1.08–1.53; agricultural—employee AOR = 0.69, 95% CI; 0.52–0.91), husbands’ occupation (agricultural—self-employed AOR = 1.48; 95% CI; 1.11–1.98), religion (other Christians AOR = 0.80; 95% CI; 0.65–0.98), economic status (richest AOR = 0.59; 95% CI; 0.47–0.73), marital status (currently married AOR = 0.44; 95% CI; 0.39–0.51), and parity (≥5 AOR = 1.85; 95% CI; 1.45–2.35). When it came to the community-level factors, region (Plateau AOR = 5.24; 95% CI; 3.18–8.64, Mono AOR = 4.12; 95% CI; 2.39–7.08) and community literacy level (high AOR = 0.69; 95% CI; 0.51–0.94) were the two main identified factors.

In Table 1 and Supplementary Material 3, the values of AIC and BIC showed that there was a considerable improvement in each of the models over the preceding model, and this confirms the goodness of fit of the final model developed in the analysis. Hence, the complete model, which incorporated the individual- and community-level factors, was chosen for its significance in affecting access to healthcare services. The empty model (Table 1 and Supplementary Material 3) showed a statistically significant variation in the odds of problems in access to healthcare service across the clusters (σ2 = 0.94, 0.80–1.10). The empty model showed that 22% of the total variance in problems to accessing healthcare services was attributed to between-cluster variations (ICC = 0.22). The between-cluster variations decreased by 1% in model 1, from 22% in the empty model to 21% in the individual-level only model. From model 1, the ICC declined to 14% (ICC = 0.14) in the community-level only model. However, it raised by 3% in the complete model (model 3, ICC = 0.17), which had both the individual- and community-level factors. This explains that the variations in the likelihood of encountering problems to accessing healthcare services could be attributed to the variances within clusters at the primary sampling units. 

## 4. Discussion

In this study we highlighted the problems in access to healthcare services and their correlates in Benin, using the 2017–2018 Benin Demographic and Health Survey. We found that 60.4% of the women had problems with accessing healthcare services for at least one reason. We found that husbands’ educational level had a significant association with problems in accessing healthcare services. Our results coincide with previous studies in Bangladesh [33] and Myanmar [23]. The plausible reason for better access to healthcare services among women with educated husbands might be due to better participation and engagement of husbands in their families’ health [34]. Women whose partners are educated are also likely to be informed regarding their fundamental human rights and may have higher health literacy. As a result, they are more likely to deal with any form of barrier to healthcare, compared to their counterparts who are less educated and may have lower health literacy [35]. Besides, regardless of women’s educational status, it is documented that husbands’ education alone significantly affects access and utilization of health services, such as uptake of children immunization, as documented in rural Haiti [36] and seven other countries (Democratic Republic of Congo, Indonesia, India, Pakistan, Nigeria, Jordan, and Ethiopia) [37]. The other plausible justification for less problems among women with educated husbands could be the fact that there is a strong association between education and income and wealth [38]. Education is the prominent factor of higher employment opportunities, earning and individual, household, and national economic growth [39,40,41], that may in turn increase accessibility for healthcare services [19,42]. Household economic status largely affects the propensity of problems to accessing health services. Scholars have documented that accessibility of health services is often influenced by financial capacity of the households, because both direct costs, like payments for drugs and services, and indirect costs, such as transport cost and unpaid working hours, negatively affect accessibility [19,42]. Out-of-pocket expenditures for healthcare are frequently the most unfair category of financing, as the poorest suffer the most. Thus, it serves as a barrier to healthcare by depriving individuals’ financial security at the point of care [3]. A large portion of Beninese have poor access to health services [13]. About 37.7% of their health expenditure is paid as out-of-pocket, in which the majority of these payments are made in the private health sector [13].

There has been a growing emphasis not only on financial bottlenecks to healthcare access, but on the economic implications of healthcare financing as well [43]. These implications comprise increased expenses for households, selling assets, or seeking financial aid from others, and each of these have the potency to bring about poverty and longer-term debt [3,44].

We further found that women and husband’s occupation were significantly associated with problems in accessing health services. More specifically, we found that women who, themselves, and women whose husband’s occupation type was agricultural—self-employed were more likely to have problems in access to healthcare services. It is noteworthy that half of the global labor forces are working in the agricultural sector [45]. Further, the developing countries account for the majority of these agricultural workers, and most of them are small-scale farmers [45].

The economy of Benin is much dependent on agriculture, as 56% of the population work in this sector [10]. Unlike workers of other sectors, agricultural workers do not usually benefit from technological advancement [45]. Women in agriculture have an increased likelihood of injuries/diseases, and they have limited access to health services [45]. Most of them lack formal education, training, or access to information about work-related risks [45]. Premature deliveries, miscarriages, and spontaneous abortions have been linked to greenhouse-associated work [45]. Despite high odds of problems observed among agricultural—self-employed women, we also noted less odds of problems in accessing healthcare service among women whose occupation was agricultural—employees in the study. The possible justification might be due to having higher health insurance among agricultural—employee than agricultural—self-employed [46].

Self-employed and employee or dependent workers may differ in terms of economic capacities. An empirical analysis in USA showed that the mean incomes of employees are higher than self-employed; nonetheless, the distribution of self-employment earning shows higher dispersal and more skewed, compared to employee [47]. Being rich or having better income, on the other hand, facilitate accessing healthcare services related to ability to pay for transport and other health services and medication [48]. Another possible reason for differences related to problems in accessing healthcare service between self-employed and employee might be due to opportunity costs [48]. Even if more potential exists in control of working time among self-employed, loss of earning and productivity are seen among self-employed, due to absence from the workplace. Being busy in managerial and organizational responsibilities, even after working time, might result in them not being seen by healthcare providers at health facilities [49]. 

We found that religion had a statistically significant association with problems in access to healthcare services. Health and matters of religion are interlinked, especially in the African context, as some illnesses have been assigned with spiritual connotations [50]. In many parts of Africa, religion is deemed crucial to life, and hence the positive virtues of religion should be optimally utilized to enhance women’s health [51]. Religion can be used as a means of controlling human action and behavior [51]. Religious dogmas can cause a number of African women to forgo some vital maternal health services, refuse services by male health personnel, and choose faith-based approach over quality medicine [45]. In most religions, generally positive features are practiced for building health, with no restrictions for seeking medical help. For example, in Muslim believers, there is mandatory washing of hand, arms, face, and feet. Consumption of alcohol and other intoxicants are forbidden; smoking and using other substances that potentially harm the body are frowned upon. Healthcare providers are considered as God’s/Allah’s agents for healing. Overall, though it varies based on cultures, many Muslims believe in holistic healthcare, and leading a healthy life is considered as a religious obligation [52].

In Christians, for instance in Eastern Orthodox, followers highly support medical care because of the belief that the medical art has been given to them by God, who directs their whole life, as a model for the cure of the soul [52]. Since all healing comes from God, they consider healthcare providers as healing administrators. From Christians, Protestant followers, for instance, focus on benefits of individual wellbeing and relationships, and encourage health practices that support their mind, body, and spirit [52]. The Catholic church understands that treating individuals as human beings and caring for all aspects of their being is necessary to living the Gospel [32,52]. 

Regarding Voodoo, the peoples who follow such belief perceive that whatever good or bad things happening are because of the impulses of spirits [53]. Unlike most other religion followers, the fundamental concepts for cause of illness are considered as supernatural or natural in Voodoo beliefs. For instance, if the spirits are sad with you, they can make you sick [53]. This may become a barrier to the followers to practicing disease prevention activities, and to delay health-seeking behavior or not to seek at all. Therefore, intensive and continuous appropriate health education about how disease happens are required to enhance health-seeking behavior [53]. Evidence from Uganda showed that teamwork between religious leaders and health administrators is vital for reducing misperception and false beliefs that may not actually be of the religion’s view, that in turn increase uptake of health services [54]. As a result, working closely with religious leaders may have positive outputs to increase accessibility and utilization of maternal health services [55].

Marital status also had a significant association with access to healthcare services, with married women reporting better access than non-married women. This might be due to the fact that marriage has “spare capacity”—the capacity to commit one’s precious time, energy, and resources for healthcare due to division of labor and allocated tasks in the home [56]. Also, marriage enables resource allocation and investment on mutual basis [57]. The self-selection nature of marriage, commonly known as “marriage selection,” is subsequent from unseen traits that affect healthcare access, utilization, and outcome [57,58,59]. Unmarried women are less likely to access resources that later influence them to have health insurance, and disposable income that in turn affects access and utilization of health services [60,61]. Evidence shows that being married is prognostic of better health [62,63,64], and may be attributable for proper access and utilizations. There is a positive relation between marriage and health [57]. This protective role of strong relationship among spouses (mainly women) for health could be as a result of caretakers, and provision of physical and emotional support [65].

We found that the odds of multipara women to encounter problems to accessing healthcare services were high. This could be related to previous unsatisfactory experience at health facilities [66]. Mothers with lower parity usually are vigilant about pregnancy, delivery, and related health conditions, and as a result are highly likely to seek and use healthcare services [67].

Conversely, mothers with higher parity have more knowledge and experience from their previous pregnancies, delivery, and related conditions, and they develop confidence and perceive that healthcare may not be as compulsory [68]. Moreover, women with high parity are usually challenged by management of families and many children at home, and insufficient resources related to large family size [69]. 

The magnitude of problems in access to healthcare service varied across regions in Benin. Similar findings have been reported by Paul et al. [70]. Some scholars also reported irregular patterns of maternal healthcare utilization across regions in Benin, suggesting the possibility of peculiar and yet distinct health barriers across regions [71]. As of 2019, more than half of the population (52.14%) across the regions were in rural settlements [72]. Evidence shows variation in health service accessibility and utilization, as well as expenditure, not only among individuals, because it also happens across regions within a country [20,24,73]. Disparities in the acceptability and quality of healthcare service across health facilities in different regions might explain the variation [19]. The other justification for the difference in healthcare access problems across regions might be by organization-related reasons, such as differences in treatment by doctors, nurses, and other health professionals, [74] and others, such as disparities in shortage of healthcare providers [22].

We found that community literacy level was significantly associated with problems in access to healthcare services. Women in communities with better literacy encountered less challenges in accessing health services. This is mostly associated with income level [75]. Education is an indispensable element for increasing the health and overall wellness of persons. It actually aids in promoting and sustaining wholesome lifestyles and positive selections, thereby augmenting human development as a whole [75]. Parental education impacts neighborhood choice through income, aspirations, and lifestyle [75].

### Strengths and Limitations of the Study

In this study, we used a multi-model approach to investigate a broad range of individual- and community-level factors associated with access to healthcare services in Benin. We used the most updated nationally representative data, which contributed to understanding the current barriers and facilitators for achievement of universal health coverage.

However, the study needs to be seen with the following two limitations: first, the cross-sectional design of the study did not allow us to ascertain the cause–effect relationship; second, we excluded two factors, attitude and quality of services, that required qualitative studies to explain them (despite our attempt to incorporate multiple factors from the DHS dataset).

## 5. Conclusions

Overall, three-fifths of women in the reproductive age encountered problems in accessing healthcare services in Benin. Husband’s education, women’s occupation, husband’s occupation, religion, economic status, marital status, and parity were significant individual-level factors. Region and community literacy level were the two main identified community-level factors. Policies should not only target empowering women and men through education and economy, they should also focus on increasing the communities’ literacy level through ensuring equity-based national education coverage. Moreover, working with religious leaders and giving priority to some regions with poor access to healthcare services would be necessary. Providing more focus on multipara and unmarried women during interventions such as counseling is also worth considering.

## Figures and Tables

**Table 1:** Multilevel multivariable logistic regression results for healthcare access problems and its associated factors among women in the reproductive age groups: Evidence from 2017/18 Benin Demographic and Health Survey.

Characteristics	Model III
**Age (Years)**	
15–19	
20–24	1.16 (0.93–1.44)
25–29	0.95 (0.76–1.19)
30–34	0.83 (0.65–1.06)
35–39	0.79 (0.61–1.02)
40–44	0.85 (0.65–1.12)
45–49	0.83 (0.62–1.10)
**Women’s educational status**	
No formal education	
Primary	0.98 (0.86–1.12)
Secondary	0.91 (0.78–1.06)
Higher	0.76 (0.49–1.17)
**Husband’s educational status**	
No formal education	
Primary school	1.11 (0.98–1.27)
Secondary school	0.94 (0.82–1.08)
Higher	0.70 (0.55–0.89) **
**Women’s occupation**	
Not working	
Professional/technical/managerial	0.91 (0.68–1.21)
Sales	1.16 (1.00–1.34) *
Agricultural—self employed	1.28 (1.08–1.53) **
Agricultural—employee	0.69 (0.52–0.91) **
Services	1.28 (1.07–1.52) **
Skilled manual	0.98 (0.82–1.18)
Other unclassified	1.13 (0.79–1.61)
**Husband’s occupation**	
Not working	
Professional/technical/managerial	1.07 (0.79–1.45)
Sales	1.39 (1.02–1.91) *
Agricultural—self employed	1.48 (1.11–1.98) **
Agricultural—employee	1.11 (0.75–1.64)
Services	1.31 (0.96–1.77)
Skilled manual	1.24 (0.92–1.68)
Other unclassified	1.19 (0.83–1.71)
**Religion**	
Vodoun (ref)	
Islam	0.81 (0.63–1.03)
Catholic	0.86 (0.70–1.06)
Protestant Methodist	1.02 (0.77–1.35)
Other Protestants	1.03 (0.76–1.40)
Celestes	1.06 (0.83–1.35)
Other Christians	0.80 (0.65–0.98) *
Other religions	0.58 (0.40–0.83) **
No religion	1.02 (0.78–1.33)
**Wealth quintiles**	
Poorest	
Poorer	0.97 (0.83–1.13)
Middle	0.90 (0.77–1.07)
Richer	0.88 (0.74–1.06)
Richest	0.59 (0.47–0.73) ***
**Ethnicity**	
Adja and related	
Bariba and related	0.75 (0.52–1.07)
Dendi and related	1.31 (0.88–1.94)
Fon and related	1.00 (0.78–1.27)
Yoa, Lokpa and related	1.14 (0.73–1.79)
Betamaribe and related	1.42 (0.93–2.15)
Peulh and related	1.40 (0.96–2.05)
Yoruba and related	0.89 (0.65–1.21)
Other Beninois	1.02 (0.67–1.55)
Other nationalities	0.67 (0.44–1.03)
**Marital status**	
Not currently married	
Married	0.44 (0.39–0.51) ***
**Parity**	
No	
1–2	1.34 (1.10–1.65) **
3–4	1.72 (1.37–2.14) ***
≥5	1.85 (1.45–2.35) ***
**Place of residence**	
Urban	
Rural	1.02 (0.82–1.26)
**Region**	
Alibori	
Atacora	2.28 (1.44–3.59) ***
Atlantic	2.97 (1.88–4.70) ***
Borgou	2.60 (1.76–3.85) ***
Collines	1.55 (0.98–2.45)
Couffo	0.83 (0.50–1.40)
Donga	1.37 (0.86–2.17)
Littoral	2.45 (1.50–4.00) ***
Mono	4.12 (2.39–7.08) ***
Oueme	3.33 (2.07–5.35) ***
Plateau	5.24 (3.18–8.64) ***
Zou	2.02 (1.27–3.23) **
**Community literacy level**	
Low	
Medium	0.72 (0.57–0.91) **
High	0.69 (0.51–0.94) *
**Community socioeconomic level**	
Low	
Moderate	0.97 (0.76–1.23)
High	0.80 (0.57–1.10)
**Random effect result**	
PSU variance (95% CI)	0.67 (0.55–0.82)
ICC	0.17
LR Test, *p*-value	χ2 = 528.30, *p* < 0.001
Wald chi-square and *p*-value	χ2 = 637.60, *p* < 0.001
**Model fitness**	
Log-likelihood	−6579.99
AIC	13,298
BIC	13,803.14
PSU	555
N	11,170

* *p* < 0.05, ** *p* < 0.01, *** *p* < 0.0001, ref: reference, AIC: Akaike information criterion, BIC: Bayesian information criterion.

## Supplementary Materials

The following are available online at https://www.mdpi.com/1660-4601/18/2/750/s1, Supplementary Material 1: Frequency distribution of study participants in health care access: Evidence from 2017/18 Benin demographic and health survey. Supplementary Material 2: Magnitude of health care access problem across explanatory variables among reproductive women: Evidence from 2017/18 Benin demographic and health survey. Supplementary Material 3: Multilevel multivariable logistic regression results for health care access problems and its associated factors among women in the reproductive age groups: Evidence from 2017/18 Benin demographic and health survey.
