# Supplementary material for: Breaking Barriers to Healthcare Access: A Multilevel Analysis of Individual- and Community-Level Factors Affecting Women’s Access to Healthcare Services in Benin
Source: Int J Environ Res Public Health. 2021 Jan 17;18(2):750. doi: 10.3390/ijerph18020750 (PMC7830614; doi:10.3390/ijerph18020750)
Supplement: Supplementary file 1 [file ijerph-18-00750-s001.pdf]

Supplementary material 1. Frequency distribution of study participants in health care access: Evidence from 2017-2018 Benin demographic and health survey.

Characteristics	N	(%)
**Age (years)**		
15-19	3,335	(20.94%)
20-24	2,916	(18.31%)
25-29	2,971	(18.65%)
30-34	2,195	(13.78%)
35-39	1,905	(11.96%)
40-44	1,333	(8.37%)
45-49	1,273	(7.99%)
**Women's educational status**		
No education	8,762	(55.01%)
Primary	3,116	(19.56%)
Secondary	3,685	(23.14%)
Higher	365	(2.29%)
**Husband educational status**		
No formal education	6,321	(56.59%)
Primary school	2,130	(19.07%)
Secondary school	2,063	(18.47%)
Higher	656	(5.87%)
**Women occupation**		
Nott working	3,527	(22.14%)
Professional/technical/managerial	543	(3.41%)
Sales	4,537	(28.48%)
Agricultural - self employed	2,574	(16.16%)
Agricultural - employee	491	(3.08%)
Services	1,842	(11.56%)
Skilled manual	1,825	(11.46%)
Other unclassified	589	(3.70%)
**Husband occupation**		
Not working	5,040	(31.64%)
Professional/technical/managerial	1,491	(9.36%)
Sales	1,007	(6.32%)
Agricultural - self employed	4,493	(28.21%)
Agricultural - employee	322	(2.02%)

Services	1,385	(8.70%)
Skilled manual	1,820	(11.43%)
Other unclassified	370	(2.32%)
**Religion**		
Vodoun	1,332	(8.36%)
Islam	4,677	(29.36%)
Catholic	3,945	(24.77%)
Protestant Methodist	786	(4.93%)
Other protestants	610	(3.83%)
Celestes	1,238	(7.77%)
Other Christians	2,205	(13.84%)
Other religions	302	(1.90%)
No religion	833	(5.23%)
**Wealth quintiles**		
Poorest	2,856	(17.93%)
Poorer	2,976	(18.68%)
Middle	2,985	(18.74%)
Richer	3,281	(20.60%)
Richest	3,830	(24.05%)
**Ethnicity**		
Adja and related	2,174	(13.65%)
Bariba and related.	1,729	(10.86%)
Dendi and related.	838	(5.26%)
Fon and related.	5,745	(36.07%)
Yoa, lokpa and relate	441	(2.77%)
Betamaribe and relate	1,065	(6.69%)
Peulh and related	1,243	(7.80%)
Yoruba and related.	1,936	(12.15%)
Other beninois	533	(3.35%)
Other nationalities	224	(1.41%)
**Marital status**		
Not currently married	7,100	(44.58%)
Married	8,828	(55.42%)
**Parity**		
No	4,172	(26.19%)

1-2	3,995	(25.08%)
3-4	3,568	(22.40%)
≥5	4,193	(26.32%)
**Place of residence**		
Urban	7,045	(44.23%)
Rural	8,883	(55.77%)
**Region**		
Alibori	1,697	(10.65%)
Atacora	1,392	(8.74%)
Atlantic	1,702	(10.69%)
Borgou	1,765	(11.08%)
Collines	1,403	(8.81%)
Couffo	1,012	(6.35%)
Donga	964	(6.05%)
Littoral	1,415	(8.88%)
Mono	816	(5.12%)
Oueme	1,260	(7.91%)
Plateau	952	(5.98%)
Zou	1,550	(9.73%)
**Community literacy level**		
Low	5,343	(33.54%)
Medium	5,344	(33.55%)
High	5,241	(32.90%)
**Community socioeconomic level**		
Low	6,724	(42.21%)
Moderate	3,916	(24.59%)
High	5,288	(33.20%)

Supplementary material 2- Magnitude of health care access problem across explanatory variables among reproductive women: Evidence from 2017/18 Benin demographic and health survey.

Characteristics	Health care access problem	Chi-square, p-value
**Age (years)**	N (%)	
15-19	1,941 (58.20%)	
20-24	1,740 (59.67%)	
25-29	1,781 (59.95%)	
30-34	1,320 (60.14%)	
35-39	1,140 (59.84%)	
40-44	851 (63.84%)	
45-49	809 (63.55%)	$\chi 2$=19.41, p<0.01
**Women's educational status**		
No formal education	5,632 (64.28%)	
Primary school	1,900 (60.98%)	
Secondary school	1,917 (40.6%)	
Higher	133 (36.44%)	$\chi 2$=250.36, p<0.001
**Husband's educational status**		
No formal education	3,991 (63.14%)	
Primary school	1,376 (64.60%)	
Secondary school	1,133 (54.92%)	

Higher	243 (37.04%)	χ2=211.00, p<0.001
**Women's occupation**		
Not working	1,837 (52.08%)	
Professional/technical/managerial	221 (40.70%)	
Sales	2,791 (61.52%)	
Agricultural - self employed	1,859 (72.22%)	
Agricultural – employee	258 (52.55%)	
Services	1,168 (63.41%)	
Skilled manual	1,098 (60.16%)	
Other unclassified	350 (59.42%)	χ2=361.63, p<0.001
**Husband's occupation**		
Not working	2,988 (59.29%)	
Professional/technical/managerial	722 (48.42%)	
Sales	570 (56.60%)	
Agricultural - self employed	2,995 (66.66%)	
Agricultural – employee	182 (56.52%)	
Services	816 (58.92%)	
Skilled manual	1,103 (60.60%)	
Other unclassified	206 (55.68%)	χ2=177.71, p<0.001
**Religion**		

Vodoun	914 (68.62%)	
Islam	2,579 (55.14%)	
Catholic	2,269 (57.52%)	
Protestant Methodist	461 (58.65%)	
Other protestants	393 (64.43%)	
Celestes	857 (69.22%)	
Other Christians	1,330 (60.32%)	
Other religions	174 (57.62%)	
No religion	605 (72.63%)	$\chi2$=203.09, p<0.001
**Wealth quintiles**		
Poorest	2,023 (70.83%)	
Poorer	1,940 (65.19%)	
Middle	1,880 (62.98%)	
Richer	1,940 (59.13%)	
Richest	1,799 (46.97%)	$\chi2$=456.46, p<0.001
**Ethnicity**		
Adja and related	1,314 (60.44%)	
Bariba and related	856 (49.51%)	
Dendi and related	406 (48.45%)	
Fon and related	3,539 (61.60%)	

Yoa, lokpa and related	246 (55.78%)	
Betamaribe and related	810 (76.06%)	
Peulh and related	858 (69.03%)	
Yoruba and related	1,145 (59.14%)	
Other beninois	311 (58.35%)	
Other nationalities	97 (43.30%)	$\chi 2=319.54$, $p<0.001$
**Marital status**		
Not currently married	4,421 (62.27%)	
Married	5,161 (58.46%)	$\chi 2=23.78$, $p<0.001$
**Parity**		
No	2,332 (55.90%)	
1-2	2,292 (57.37%)	
3-4	2,184 (61.21%)	
≥5	2,774 (66.16%)	$\chi 2=109.17$, $p<0.001$
**Place of residence**		
Urban	3,950 (56.07%)	
Rural	5,632 (63.40%)	$\chi 2=88.16$, $p<0.001$
**Region**		
Alibori	824 (48.56%)	
Atacora	996 (71.55%)	

Atlantic	1,138 (66.86%)	
Borgou	1,123 (63.63%)	
Collines	755 (53.81%)	
Couffo	532 (52.57%)	
Donga	476 (49.38%)	
Littoral	680 (48.06%)	
Mono	610 (74.75%)	
Oueme	820 (65.08%)	
Plateau	727 (76.37%)	
Zou	901 (58.13%)	$\chi2=584.82$, $p<0.001$
**Community literacy level**		
Low	3,595 (67.28%)	
Medium	3,270 (61.19%)	
High	2,717 (51.84%)	$\chi2=266.83$, $p<0.001$
**Community socioeconomic level**		
Low	4,452 (66.21%)	
Moderate	2,406 (61.44%)	
High	2,724 (51.51%)	$\chi2=270.35$, $p<0.001$

Supplementary Material 3. Multilevel multivariable logistic regression results for health care access problems and its associated factors among women in the reproductive age groups: Evidence from 2017/18 Benin demographic and health survey.

Characteristics	Model 0	Model I	Model II	Model III
**Age (years)**				
15-19	Reference			
20-24		1.16 (0.93-1.44)		1.16 (0.93-1.44)
25-29		0.95 (0.75-1.19)		0.95 (0.76-1.19)
30-34		0.82 (0.64-1.06)		0.83 (0.65-1.06)
35-39		0.78 (0.60-1.01)		0.79 (0.61-1.02)
40-44		0.84 (0.64-1.11)		0.85 (0.65-1.12)
45-49		0.81 (0.61-1.08)		0.83 (0.62-1.10)
**Women's educational status**				
No formal education	Reference			
Primary		0.97 (0.85-1.11)		0.98 (0.86-1.12)
Secondary		0.88 (0.76-1.03)		0.91 (0.78-1.06)
Higher		0.73 (0.47-1.13)		0.76 (0.49-1.17)
**Husband's educational status**				
No formal education	Reference			
Primary school		1.12 (0.99-1.28)		1.11 (0.98-1.27)
Secondary school		0.93 (0.81-1.07)		0.94 (0.82-1.08)
Higher		0.70(0.55-0.89)**		0.70 (0.55-0.89)**
**Women's occupation**				
Not working	Reference			
Professional/technical/managerial		0.92 (0.68-1.23)		0.91 (0.68-1.21)
Sales		1.16 (1.01-1.35)*		1.16 (1.00-1.34)*
Agricultural-self employed		1.26 (1.06-1.50)**		1.28 (1.08-1.53)**

Agricultural–employee		0.68(0.51-0.89)**		0.69 (0.52-0.91)**
Services		1.28(1.07-1.53)**		1.28 (1.07-1.52)**
Skilled manual		1.00 (0.83-1.19)		0.98 (0.82-1.18)
Other unclassified		1.11 (0.78-1.59)		1.13 (0.79-1.61)
**Husband occupation**				
Did not work	Reference			
Professional/technical/managerial		1.06 (0.78-1.44)		1.07 (0.79-1.45)
Sales		1.36 (1.00-1.86)*		1.39 (1.02-1.91)*
Agricultural - self employed		1.48 (1.11-1.98)		1.48 (1.11-1.98)**
Agricultural-employee		1.06 (0.72-1.57)		1.11 (0.75-1.64)
Services		1.29 (0.95-1.76)		1.31 (0.96-1.77)
Skilled manual		1.25 (0.93-1.69)		1.24 (0.92-1.68)
Other unclassified		1.20 (0.83-1.73)		1.19 (0.83-1.71)
**Religion**				
Vodoun	Reference			
Islam		0.79 (0.62-1.01)		0.81 (0.63-1.03)
Catholic		0.86 (0.70-1.06)		0.86 (0.70-1.06)
Protestant Methodist		1.04 (0.79-1.38)		1.02 (0.77-1.35)
Other protestants		1.08 (0.80-1.47)		1.03 (0.76-1.40)
Celestes		1.10 (0.86-1.40)		1.06 (0.83-1.35)
Other christians		0.80 (0.65-0.98)*		0.80 (0.65-0.98)*
Other religions		0.62 (0.43-0.89)*		0.58 (0.40-0.83)**
No religion		1.04 (0.80-1.36)		1.02 (0.78-1.33)
**Wealth quintiles**				
Poorest	Reference			
Poorer		0.95 (0.81-1.11)		0.97 (0.83-1.13)
Middle		0.87 (0.73-1.02)		0.90 (0.77-1.07)
Richer		0.83(0.70-0.99)*		0.88 (0.74-1.06)
Richest		0.53(0.43-0.66)***		0.59 (0.47-0.73)***
**Ethnicity**				

Adja and related	Reference			
Bariba and related		0.82 (0.61-1.10)		0.75 (0.52-1.07)
Dendi and related		1.30 (0.92-1.83)		1.31 (0.88-1.94)
Fon and related		1.17 (0.96-1.44)		1.00 (0.78-1.27)
Yoa, lokpa and related		1.14 (0.77-1.69)		1.14 (0.73-1.79)
Betamaribe and related		1.64(1.16-2.32)**		1.42 (0.93-2.15)
Peulh and related		1.68(1.21-2.33)**		1.40 (0.96-2.05)
Yoruba and related		1.14 (0.87-1.48)		0.89 (0.65-1.21)
Other beninois		1.00 (0.69-1.44)		1.02 (0.67-1.55)
Other nationalities		0.75 (0.50-1.12)		0.67 (0.44-1.03)
**Marital status**				
Not currently married	Reference			
Married		0.43(0.38-0.49)***		0.44 (0.39-0.51)***
**Parity**				
No	Reference			
1-2		1.35(1.10-1.65)**		1.34 (1.10-1.65)**
3-4		1.73(1.38-2.16)***		1.72 (1.37-2.14)***
≥5		1.87(1.47-2.37)***		1.85 (1.45-2.35)***
**Place of residence**				
Urban	Reference			
Rural			0.98 (0.81-1.19)	1.02 (0.82-1.26)
**Region**				
Alibori	Reference			
Atacora			2.81(1.93-4.09)***	2.28 (1.44-3.59)***
Atlantic			3.65 (2.56-5.20)***	2.97 (1.88-4.70)***
Borgou			2.17 (1.54-3.07)***	2.60 (1.76-3.85)***
Collines			1.58 (1.09-2.27)*	1.55 (0.98-2.45)
Couffo			1.23 (0.85-1.79)	0.83 (0.50-1.40)

Donga			1.12 (0.75-1.66)	1.37 (0.86-2.17)
Littoral			2.31 (1.55-3.42)***	2.45 (1.50-4.00)***
Mono			4.81 (3.19-7.25)***	4.12 (2.39-7.08)***
Oueme			3.56 (2.46-5.16)***	3.33 (2.07-5.35)***
Plateau			4.36 (2.94-6.46)***	5.24 (3.18-8.64)***
Zou			2.038 (1.42-2.91)***	2.02 (1.27-3.23)**
**Community literacy level**				
Low	Reference			
Medium			0.69 (0.56-0.86)**	0.72 (0.57-0.91)**
High			0.54 (0.41-0.71)***	0.69 (0.51-0.94)*
**Community socioeconomic level**				
Low	Reference			
Moderate			0.84 (0.68-1.03)	0.97 (0.76-1.23)
High			0.52 (0.40-0.69)***	0.80 (0.57-1.10)
**Random effect result**				
PSU variance (95% CI)	0.94 (0.80-1.10)	0.91 (0.76-1.09)	0.57 (0.48-0.68)	0.67 (0.55-0.82)
ICC	0.22	0.21	0.14	0.17
LR Test, p-value	χ2=1683.82, p<0.001	χ2=791.89, p<0.001	χ2=868.20, p<0.001	χ2=528.30, p<0.001
Wald chi-square and p-value	Reference	χ2=522.75	χ2=247.28, p<0.001	χ2=637.60, p<0.001
**Model fitness**				
Log-likelihood	-9867.52	-6639.58	-9762.03	-6579.99
AIC	19739.04	13385.16	19560.07	13298
BIC	19754.39	13773.18	19698.23	13803.14
PSU	555	555	555	555
N	15,928	11,170	15,928	11,170

* p<0.05, ** p<0.01, *** p<0.0001, ref: reference, AIC: Akaike Information Criterion, BIC: Bayesian Information Criterion.
